# Valorization of agro-industrial waste through solid-state fermentation: Mini review

**DOI:** 10.1016/j.btre.2024.e00873

**Published:** 2024-12-30

**Authors:** Mohammad Perwez, Sameer Al Asheh

**Affiliations:** a Special Centre for Nanoscience, Jawaharlal Nehru University, New Delhi 110067, India; b Department of Chemical and Biological Engineering, American University of Sharjah, Sharjah, P.O.Box 2666, United Arab Emirates

**Keywords:** Solid state fermentation, Bioreactor, Enzymes, Biopesticide, Antibiotics, Biofertilizer

## Abstract

Agriculture and industrial waste are produced in large volumes every year worldwide, causing serious concerns about their disposal. These wastes have high organic content, which microorganisms can easily assimilate into relevant value-added products. Valorization of agro-industrial waste is required for sustainable development. Solid state fermentation is an excellent method of utilizing waste for circular bioeconomy. Exploitation of agro-industrial waste as a substrate utilizing microorganisms for solid state fermentation provides beneficial products for use in industries and other fields. The use of waste reduces the cost of production of value-added products. This method is an environmentally friendly, economical and feasible approach for waste management. This review discusses the factors affecting the production of value-added products through solid state fermentation. It also discusses the valuable products from solid state fermentation technology, such as antibiotics, enzymes, organic acids, bioremediation, biosurfactants and biofertilizers. Challenges and future prospects are also presented.

## Highlights

• The fundamentals and advantages of SSF focusing on its sustainable application.
• Agricultural and industrial waste as substrate.
• Factors influencing value added products through solid state fermentation, such as temperature, pH, moisture, substrate selection and supplements, particle size, and inoculum size.
• Products of solid state fermentation may include Antibiotics, Enzymes, Organic acids, Bioremediation, Biosurfactants, and Biofertilizer.
• Current limitations of SSF, and potential advancements for sustainable bioprocessing and product development.

## 1 Introduction

The vast quantities of waste generated from domestic, industrial, food, and agricultural sources present a significant global challenge. In developing countries where agriculture is the main source of economic activity, agricultural waste is disposed of by burning, dumping, or unplanned landfills. It may lead to environmental pollution and cause harmful effects on animal, human, and plant lives. These untreated wastes may also lead to climate change by releasing greenhouse gases. Juice, coffee and cereal industries produce peels, coffee pulp and husks, respectively, in larger amounts and are considered as agro-industrial waste [1]. These wastes contain significant amounts of organic matter rich in sugar, which could be easily assimilated by microbes producing industrially relevant products or value-added products [2,3]. Organic waste is transformed into secondary metabolites produced by microorganisms following their growth phase. Secondary metabolites produced by microbes have no role in the growth and reproduction of microbes but are considered useful materials and are important for other secondary needs. Secondary metabolites produced by microbes include acids [4], antibiotics [5], antioxidants [6], biofuel [7], biosurfactants [8], and other secondary metabolites.

Two types of fermentations are used for the production of microbial metabolites, namely submerged fermentation (SF) and solid-state fermentation (SSF). Submerged fermentation have such advantages as online monitoring and automation while microbes are grown in natural habitat in solid state fermentation [9,10]. SSF is the cultivation process through which microbes are grown on moist substrate as a source of nutrition in the absence or near absence of free-flowing water with the continuous flow of air [10]. SSF can be considered as an alternative approach for the disposal of agricultural, domestic and industrial waste [[11], [12], [13], [14], [15]]. SSF can be used to convert substrates such as bagasse, peels of fruits and vegetables, straw, husk, and brans of cereals [16] into useful products while utilizing microbes such as bacteria, fungi, and yeast in the fermentation process. Yeast and fungi are usually safe, but some produce mycotoxins [[17], [18], [19]].

In SSF processes, different types of reactors are used, such as packed bed reactors, rotating drums, mechanically stirred reactors, tray reactors, and plug flow configurations [20,21]. Tray bioreactors, traditionally used in SSF, feature a simple, static design with unmixed beds and no forced aeration. Fermentation occurs on stationary perforated trays to allow passive aeration, but only thin substrate layers are used to prevent overheating and maintain aerobic conditions [22]. Khanahmadi et.al. maximized the synthesis of xylanase enzyme by SSF in a tray bioreactor using *Aspergillus niger* CCUG33991 with wheat bran serving as a low-cost, hemicellulosic agro-waste and an ideal carbon and energy source [23]. Packed-bed bioreactors have gained significant attention for their ease of use in converting lignocellulosic biomass. In these systems, the substrate bed remains stationary during fermentation while air is pushed through the gaps between particles to reach the outlet. Some designs, known as "intermittently mixed packed-bed bioreactors," involve occasional mixing to ensure uniformity, add water, or adjust flow paths. The minimal mixing or lack thereof makes them ideal for cultivating filamentous fungi, which are sensitive to frequent agitation due to potential damage to their hyphae. Despite this, packed-bed bioreactors can also be effectively used with unicellular microorganisms [24]. The packed bed bioreactor with agro- industrial waste (wheat bran, sugarcane bagasse and orange pulp and peel) was utilised for the production of cellulase enzyme which helped in analysis of structural properties of the packed bed like moisture content, packing technique, microscopic analysis of cell size, and porosity along the fermentation was determined [25].

**Fig. 1:**
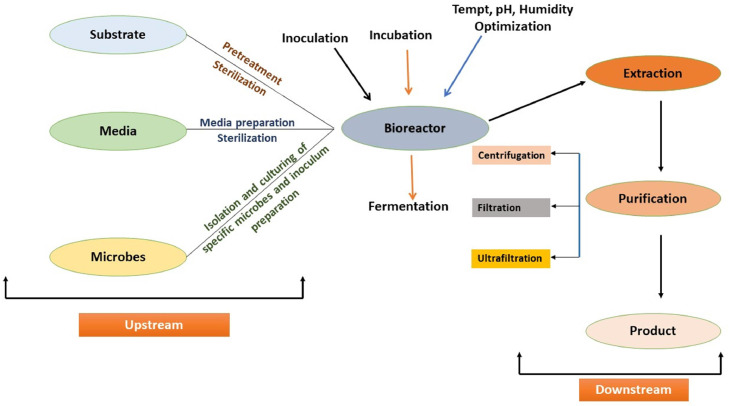
Upstream and Downstream processes involved in valorization of agro-industrial waste through solid state fermentation.

Three major steps are involved in solid state fermentation: upstream, midstream and downstream. The upstream process involves the pretreatment and sterilization of substrate, media preparation, and microbe inoculum preparation. Midstream involves the process of SSF where microbes are incubated with substrate and the optimization of different conditions are monitored. In the downstream processing the product formed are extracted, purified and then packaged [26]. These stages are summarized in Fig. 1. SSF is preferred over SF due to lower energy consumption, less chances of contamination and less complex machinery involved. The problem associated with the reactor operation for the scale up process is the mass transfer limitations and heat transfer to organic matter due to the solid state which could harm the strain [27] due to rise in temperature. This makes it difficult to use SSF at large scale for commercial purpose [28].

The present review discusses the use of microorganisms, the substrate required, the process optimization approach, and secondary metabolites produced during SSF. The challenges faced by SSF in scaling up production, prospects, and the use of emerging technologies to produce secondary metabolites efficiently are also presented.

## 2 Solid state fermentation

The cultivation of solid natural substrate provides matrix for support and nutrition for microbial growth [10,29]. Lignocellulosic agricultural residues, agro-industrial products, and by-products are ideal substrates for SSF. Different parameters affect the fermentation process such as pH, temperature, nutrient, substrate moisture content, incubation time, and inoculation volume; these parameters vary with different microbes. Therefore, it is essential to understand the microorganism's growth conditions to maximize metabolite production by SSF. The limited amount of water in SSF provides several advantages including easy recovery of product, low cost, reduced downstream processes and reduced energy requirements [30]. There are several steps involved in SSF including: (1) selection of substrate, (2) pretreatment of substrate through mechanical, chemical or biochemical processes to make the bound nutrient available for hydrolysis and fermentation, (3) hydrolysis of the pretreated substrate, (4) fermentation for obtaining desired microbial metabolite, and (5) downstream processing for purification and quantification of the product. SSF is an excellent bioprocess for producing secondary metabolite (SM) using agro-industrial waste. SM production in microorganisms begins during the idiophase, following the active growth phase known as trophophase. During Idiophase, SM production starts by means of stress condition [31]. When the nutrient required for microbial growth is drained off, microbes start producing SM, which accumulates. For example, penicillin is produced by *Penicillium chrysogenum* when glucose is not available for consumption, so it starts to feed on lactose [32]. SM is produced by microbes as those chemicals are not required by microbes for their growth and reproduction.

## 3 Agricultural and industrial waste as substrate

**Fig. 2:**
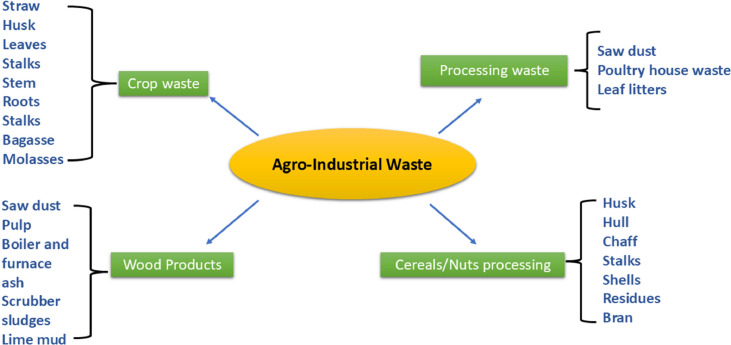
Diagram showing different kinds of solid wastes generated through agricultural activities and industrial processes.

Agricultural and industrial waste are very difficult to manage or dispose of, which leads to unplanned landfills causing hazardous impacts on the environment human and animal life. Agricultural wastes are rich in various compounds that can be used to produce biogas, biofuel, and different secondary metabolites once fermented. These waste products are available at low cost, which decreases the cost of production of these products and reduces environmental pollution. Leftovers that are generated through agricultural and industrial activities are regarded as agro-industrial waste [33]. These wastes consist of lignocellulosic components which are rich in carbon and nitrogen [34]. This lignocellulosic biomass is further hydrolyzed by different types of enzymes produced by various microorganisms [35]. The different kinds of solid wastes are illustrated in Fig. 2**.**

Agricultural wastes can be either process residues or field residues. After crop harvesting, the residues left in the field are called field residues. The residues remaining after the crop is processed into valuable products are called process residues. Field residues may include stems, leaves, and seeds, while process residues are husks, pulp, roots, peel, straw, etc. These residues can be further utilized for other purposes or can be used as animal feed, fertilizers, etc. The food processing industries, such as those involved in juice, chips, meat, confectionery, fruits, and vegetables, generate substantial amounts of organic waste and effluents each year. Food industry waste constitutes different components such cellulose, hemicellulose, lignin and moisture. These constituents can easily be digested by microbes, which produce value-added products like bioethanol and biogas. In India, waste from fruits and vegetables amounts to 20% of the original products [36]. The untreated waste produced from the juice industries contains a high value of BOD (Biochemical Oxygen Demand refers to the amount of dissolved oxygen needed by microorganisms to decompose the organic matter present in wastewater under aerobic conditions) and COD (Chemical Oxygen Demand is a measure of the total quantity of oxygen required to chemically oxidize both organic and inorganic substances in a water sample) and suspended solids which remain unutilized. This waste can also adversely affect the environment, human and animal life forms.

## 4 Factors influencing value added products through solid state fermentation

### 4.1 Effect of temperature

Temperature is an important factor governing the microbial growth in the bioreactor. Every microorganism grows at a particular temperature range, producing secondary metabolites and enzymes [37,38]. Initially, the growth of microbes inside the bioreactor does not produce heat due to the low biomass concentration. However, as the growth of microorganisms inside the bioreactor increases, the biomass concentration increases, which increases the microbes' metabolic activity, leading to increased temperature. So, the major challenge associated with the SSF is the production of heat and mass transfer difficulty, which affects the growth of microbes and products formed by microbes [39]. Heat generated leads to loss of moisture in larger reactors, which affects the growth and the product yield. It also leads to condensation, which increases the amount of water in the fermented matrix, promoting heterogeneity and, thus, difficulty maintaining a homogenous ideal temperature, affecting growth and SSF process [39]. If heat production rate exceeds the heat removal rate, then the temperature increases. Aeration is needed to remove the hot air from the vent. Heating can also be prevented by agitation and aeration. Cephalosporin C was produced from *Acremonium chrysogenum* at an optimum temperature of 30 °C and resulted in an antibiotic yield of 5596 µg/g, while at temperatures other than the optimal, there was less yield reported [40]. *Trichoderma asperellum* strain R, *Trichoderma atroviride* strain Ta13, *Trichoderma harzianum* strain T-22 (ATCC 20847), and *Trichoderma reesei* strain RUT-C30 (ATCC 56765) were grown on slants of potato dextrose agar at 26 °C to produce a maximum biomass yield of 689.80 ± 80.53 mg mycelium/g substrate [41]. The production of citric acid using *Aspergillus niger* NRRL 567 grown on peat moss was analyzed in a column bioreactor under SSF, employing a statistically designed approach. The optimal citric acid yield (123.9 g/kg) was achieved at a fermentation temperature of 32 °C, identified as the key parameter influencing citric acid production [42]. Hashemi et al. investigated the production of α-amylase by *Bacillus* sp. KR-8104 in both SSF and SF, noting differences in enzyme characteristics. For instance, raising the temperature to 45 °C resulted in a notable reduction in enzyme activity (around 20.5 %) compared to the activity at 37 °C in SF [43].

### 4.2 Effect of pH

pH is a very important parameter in SSF process as it affects the growth rate of microbes in the solid media. The major reason for the change in pH during SSF is due to the release of organic acids such as citric, lactic and acetic acid, resulting in a decrease in pH, while assimilation of organic acid causes pH to increase and urea hydrolysis leads to alkalinization. Accordingly, pH in the solid media should be adjusted and monitored when required. Using urea instead of ammonia can be used to control the pH [44]. Hydrolysis of urea releases ammonia, which controls the acidification of the SSF. Ammonium salts with urea or nitrate have been used in different ratios to prevent acidification and alkalinization [29]. Microbes have different ranges of pH required for growth; for example, bacteria grow mostly at neutral pH, filamentous fungi grows at a large pH range of 2 to 9 and optimum pH of 3.8 to 6, and yeast grows at broad pH of 2.5 to 8.5 and at optimum pH of 4 to 5. Therefore, bacterial contamination in fungi and yeast can be avoided by adjusting the pH which is not suitable for bacterial growth [45]. The metabolic activity of the microorganism is very sensitive to change as the pH changes. For example, Cephalosporin C was produced by *Acremonium chrysogenum* and the maximum production was obtained at pH 6.5; Cordycepin was produced from *Cordyceps militaris* at varying pH conditions, and it was observed that pH 5.5 was the optimum for maximum production of cordycepin [46].

### 4.3 Effect of moisture

The moisture content of the substrate is essential for the growth of microbes during SSF process. Lower moisture content reduces the solubility of the substrate and nutrient, while particle agglomeration occurs at higher moisture content, which further reduces oxygen transfer. In general, fungi and yeast require less water content than bacteria in SSF process. Content of moisture for substrate varies from 30 to 80 %, in which bacteria require about 70 % moisture while yeast and fungi require 20–70 % [37].

Water activity (A_w_) is a measure of the amount of free water in a product, the amount of unbounded water available for microbial growth. Water activity is given by P_s_/P_o_, which represents the ratio of water vapor pressure of solid substrate (P_s_) to vapor pressure of pure water (P_o_). The dehydration of the substrate or solute absorption by the substrate reduces the water activity, affecting growth of microbes, increasing lag time and affecting production [47]. Shah et al. (2005) noted that the production of xylanase, cellulase, and proteins was influenced by the composition of the moistening agent [48]. A locally isolated strain of *Trichoderma harzianum* was examined for xylanase production using lignocellulosic substrates in SSF. All media with various moistening agents supported enzyme production, but the highest xylanase yield (146 IU/ml) was achieved with distilled water as the moistening agent. In comparison, the use of tap water, MS medium, basal salt solution, or modified MS medium led to lower xylanase production [49]. SSF was carried out to assess the impact of various physicochemical parameters on the production of tannase and gallic acid by *A. heteromorphus* MTCC 8818. The study evaluated incubation time (24 to 120 h), incubation temperature (25, 30, 37, and 40 °C), pH (3.0 to 6.5), and inoculum size (0.5 % to 10 % v/v moistening agent). Different moistening agents, including TA medium and Czapek Dox medium (comprising 0.3 % NaNO_3_, 0.1 % K_2_HPO_4_, 0.05 % MgSO_4_·7H_2_O, 0.05 % KCl, and 0.001 % FeSO_4_), as well as tap water and distilled water, were tested to optimize their concentrations and effects. The moisture level (substrate to Czapek Dox medium ratios of 40 %, 50 %, 60 %, 70 %, and 80% w/v) and the addition of glucose (0.2 % to 0.6% w/v) to the sawdust were also explored. Czapek Dox medium was identified as the optimal moistening agent, leading to further optimization of its constituents to maximize enzyme production. Additionally, the effects of various salts (CaCl_2_, HgCl_2_, BaCl_2_, CdCl_2_, ZnSO_4_, CuSO_4_, MnCl_2_, and NaCl) at a concentration of 0.02% (w/v moistening agent) were investigated [50].

### 4.4 Substrate selection and supplements

Solid substrate is utilized by the microbes with adequate moisture for the optimum growth of the microbes in solid state media. Pretreatment is an essential requirement to make the composition of the substrate available for microbial growth in the media. Pretreatment includes physical, chemical and enzymatic hydrolysis to improve nutrient enhancement of the substrate. To efficiently utilize lignocellulosic materials, three pretreatment methods, namely physical, chemical, and combined physical-chemical, were applied to lignin to assess their impact on the SSF of biomass (straw, locust, and walnut shell) by *Aspergillus fumigatus* G-13 [53]. The study analyzed changes on the physical and chemical structure before and after pretreatment and fermentation. It was found that combined pretreatment yielded the highest enzyme production, with optimal conditions of 60–80 mesh particle size, a 1:1.5 solid-to-liquid ratio, and pretreatment with 8 % Ca(OH)₂ at 60 °C for 48 h. This method significantly enhanced enzyme activity, increasing it by 4.81–28.91 times for locust and 7.68–35.20 times for walnut shell while also altering the crystallinity and disrupting bonds between lignin, hemicellulose, and cellulose [51]. In another study [54], sodium hydroxide was employed for alkaline pretreatment of rice straw to enhance solid substrate preparation for laccase production using SSF. The results indicated that sodium hydroxide pretreatment improved enzymatic digestibility and water retention, promoting uniform mycelium growth and nutrient utilization during SSF. The optimal laccase production of 2912.34 U/g was achieved with pretreated rice straw (1 hour, diameter < 0.085 cm), representing a 7.72-fold increase over the control [52]. One of the studies utilizes a biological pretreatment method to enhance the quality of oat straw pellets using *Trametes versicolor* 52 J (TV52J) and *Phanerochaete chrysosporium* (PC) using SSF [53]. Cellulolytic enzyme complex was applied for enzymatic saccharification of pretreated wheat straw, highlighting the potential of the indigenously developed enzyme mix. Both acid and alkali pretreatments were tested, with alkali treatment yielding higher sugar levels compared to acid pretreatment due to compositional differences resulting from the treatments [54]. The cost of SM production depends on the kind of substrate and the supplementations provided. The selection of substrate can reduce the overall cost of the SSF. This involves an appropriate selection of agricultural, domestic and industrial waste. The composition and properties of the supplements also affect productivity. The feasibility of producing a cellulolytic enzyme complex through mixed-culture SSF using soybean hulls was supplemented with wheat bran [54]. A notable study investigating the impact of yeast extract on glucoamylase production by *A. niger* NCIM 1248 in SSF revealed that the addition of 0.5 % yeast extract led to an approximate 20 % increase in enzyme yield [55]. Coconut oil cake was utilized as a substrate for α-amylase production by *A. oryzae* under SSF, yielding 1372 U/g ds α-amylase in 24 h. Through process optimization, including adjustments to temperature (30 °C), initial moisture (68 %), and supplementation with glucose, starch (0.5 %), and peptone (1 %), the enzyme yield was significantly enhanced to 3388 U/g ds [56]. Under SSF, *Bacillus sp*. produced high levels of α-amylase (464,000 U/g dry substrate) using wheat bran as the primary substrate. The process was enhanced by supplementing with simple nutrients such as glycerol, soybean meal, and MgSO₄·7H₂O [57]. Substrate can be selected on the basis of cost, availability and heterogeneity (cellulose, lignocellulose, starch and polysaccharide) [58].

### 4.5 Particle size

The particle size of the substrate is essential for the proper growth of microorganisms. It also allows proper heat and mass transfer in the solid-state medium. The surface area and particle volume ratio defines the accessible substrate fraction and depends upon the packaging concentration of the microorganism. Substrate size creates void space, and the air in the void space affects growth. Reduced size of the substrate leads to substrate accumulation, inhibiting aeration of microorganisms. The size of the substrate varies in SSF [59]. Smaller, larger and more number of particles improve ventilation and exchange of gasses but provide smaller area for microbial action [60]. Dilipkumar et al. utilized a packed bed reactor (PBR) for inulinase production in SSF using *Kluyveromyces marxianus* with press-mud as the substrate. The authors applied response surface methodology (RSM) to optimize key process parameters, including air-flow rate, packing density, and particle size, resulting in increased inulinase production [61]. Mahanama et al. employed statistical modeling of SSF to develop a framework for quantitative and mechanistic fermentation design aimed at microbial production of Vitamin K2 using *Bacillus subtilis* in static bed tray fermentation. Notably, yield reductions caused by increased bed heights were significantly mitigated by the corresponding increase in particle size, which improved substrate porosity. The polynomial model accurately represented the experimental data [62]. The effects of rice bran particle size (0.18–0.39 mm) and ammonium sulfate concentration (2–8 g/L) on biomass production, protein, and phenolic content from SSF with *Rhizopus oryzae* (CCT 1217) were investigated. Particle size positively influenced biomass production but negatively affected protein and phenolic content, while ammonium sulfate concentration enhanced biomass and phenolic yields. Cultivating the fungus with 0.18 mm rice bran and 8 g/L ammonium sulfate resulted in protein levels of 20 g/100 g dry weight and phenolic content of 4 mg/g dry weight, showing significant increases over unfermented rice bran and demonstrating the fermentation's potential to enhance compound recovery for food formulations [63].

### 4.6 Inoculum size

Appropriate inoculum size is required to grow microbes and synthesize SM. Under optimized SSF conditions (31 °C, 42.86% moisture content, and 5.5% inoculum size), mannanase production reached 45.12 IU/mL, significantly higher than the pre-optimized conditions (30 °C, 50% moisture, and 10% inoculum size), which yielded 34.42 IU/mL [64]. A smaller inoculum size leads to fewer cells in the production medium, which requires more time to reach the optimal cell count needed to efficiently utilize the substrate and produce the desired product while as the inoculum size exceeds a limit, leads to limitation in mass transfer and reduced metabolism. In a study, *Fusarium solani* effectively degraded 1 g/L of caffeine under optimized conditions (pH 5.8, 24 °C, and an inoculum size of 4.8 × 10⁵ spores/mL), along with the accumulation of theophylline (0.33 g/L) after 120 h. While lower inoculum sizes slowed the process, increasing the inoculum to 5 × 10⁵ spores/mL enhanced biodecaffeination efficiency to 83.6 %. These findings highlight the optimal parameters for effective caffeine degradation and theophylline recovery [65]. Meroparamycin production by *Streptomyces* sp. strain MAR01 was optimized using wheat bran as a cost-effective substrate in solid-state fermentation, yielding the highest antibacterial activity and minimal residual substrate. An inoculum size of 2 mL (5 × 10⁹ spores/mL) was ideal, as lower densities led to insufficient biomass and contamination risks [66]. A solid-state fermentation (SSF) method was developed for producing postbiotics with antimicrobial, antioxidant, and anti-inflammatory properties. The optimized fermentation conditions included an initial water content of 50 %, a fermentation duration of 8 days at 37 °C, and a 1:1 ratio of *Bacillus amyloliquefaciens* J and *Lactiplantibacillus plantarum* SN4 with a total inoculum size of 8 %. The SSF medium composition was optimized to include peptide powder (4 %), wheat bran (37.4 %), corn flour (30 %), and soybean meal (28.6 %). A significant improvement in inhibition rates (*p* < 0.05) was observed as the inoculum size increased from 4 % to 8 %, while further increases to 10–12 % led to a decline in activity (*p* < 0.05) [67]. In another study on glutaminase production through 48-hour SSF, increasing inoculum size led to a gradual rise in enzyme synthesis, with maximum yields achieved using 2 mL of 48-hour-old inoculum. The highest enzyme activity was 8.53 U/gds for sesame oil cake and 3.99 U/gds for wheat bran. Further increases in inoculum size resulted in reduced enzyme production for both substrates [68]. Fermentation is carried out using mycelial or spore inoculum. Spores do not metabolize, and their metabolism starts with certain kinds of enzymes. If the density of the inoculum is higher, the risk of contamination decreases [69].

## 5 Products of solid-state fermentations

### 5.1 Antibiotics

Antibiotics are the secondary metabolites synthesised by microbes to inhibit or kill the growth of other microbes. Antibiotics attack different cells' machinery, such as replication (quinolones and fluoroquinolones), transcription (rifamycins), and translation (erythromycin and azithromycin). Other methods by which antibiotic affects bacterial cell machinery involve targeting the cell wall (penicillin and methicillin attacks peptidoglycan), cell membrane (polymyxin and amphotericin), and ribosomes (tetracycline and chloramphenicol). Antibiotics can be produced by fermentation or synthetic processes. Liquid fermentation can be used to produce antibiotics but has limitations since prolonged incubation in the submerged culture conditions leads to cell autolysis [70].

The energy requirement by SSF is quite low, and it can be used to produce antibiotics in higher concentrations [71]. SSF requires substrate (agricultural, domestic and industrial residues) and specific microbial strains for antibiotic production. Several antibiotics have been prepared in much greater yield using SSF. Corn cob, sawdust and rice hulls were used as a substrate for the production of oxytetracycline [72]. Oxytetracycline was also produced from groundnut shell as the substrate using *Streptomyces rimosus* as a microbial strain [73]. Several other researchers also produced antibiotics using SSF, such penicillin [74], iturin [75], cephalosporin C [76], cyclosporin A [77], tetracycline [73], and rifamycin [78]. Minimum energy requirement, less investment, higher productivity and fewer disadvantages make SSF more efficient for antibiotic production than SF [71].

### 5.2 Enzymes

Enzymes are the biological catalysts mostly used in industrial processes. Enzymes are highly specific towards their substrate and catalyze the reaction efficiently, producing the desired product. Production of enzymes through SSF utilizing microbes is one of the important applications of SSF process. In the previous decades, a large number of microbes have been utilized to produce enzymes for industrial purposes. Efforts have been made to produce commercially relevant enzymes at a lower cost from new microbes and microbial sources, modifying media, and engineering strains to get better yield. For example, Bran was used for glucoamylase and protease production by SSF using *Aspergillus awamori* and *Aspergillus oryzae* [79]. Sorghum-coffee pulp mixture was used to evaluate the synergistic and antagonistic effect on demethylase production using *Rhizopus oryzae* (MUCL 28168) by SSF [80]. Examples of enzymes produced by solid state fermentation may include α-amylase [43], L-asparaginase [81], cellulase [82], phytase [83], lipase [84], laccase [85], xylanase [86], protease [87], invertase [88], xylosidase [89], chitin deacetylase [90], pectinase [91], and glucoamylase [92].

#### 5.2.1 Enzymes for biofuel production

The current demand of biofuel for sustainable development, biomass saccharifying enzymes are required. SSF was used to produce cellulase from *Aspergillus niger* (ATCC 16404) strains using inexpensive agro-industrial wastes such as rice rust, rice bran, whey and sugarcane bagasse. For example, 40 units/g dry substrate of enzyme was produced by *Aspergillus niger* [115]. It was reported that 2.25 folds of enzyme yield produced by *Aspergillus niger* strain (ATCC 16404) compared to that of *Trichoderma reesei* (CCT 2768) [93]. Cellulase produced by SSF was used for bioethanol production. Cellulase produced a better-reducing sugar yield (72.2 %) compared to commercial enzymes (68.7 %) [94]. The comparison of cellulase produced by *Neurospora sitophila by* SSF and SF showed CMCase, FPA and β-glucosidase activities, which were higher (53–181 times) than that of SF [95].

Xylanolytic enzymes were also to produce biofuel. Xylanases have been produced by fungal bacterial and actinomycetes culture. Xylanolytic enzymes can hydrolyze the C-5sugars from hemicellulose fraction of biomass. Xylanase production by SSF using *Aspergillus foetidus* MTCC 4898 under optimized conditions resulted in 8450 units/g substrate of xylanase. Recent enzymes produced by SSF are listed in Table 1**.**

**Table 1:** Value added products of solid-state fermentation.

Product of SSF	Microorganism	Substrate	Yield	Ref.
**Organic Acids**				
Succinic acid	*Actinobacillus succinogenes*	Fruit and vegetable waste	1.18 g SA/g sugar	[96]
Humic acid	*Trichoderma reesei*	Oil palm fibres of empty fruit bunch	350 mg HA per 100 g of fibers	[97]^)^
Gibberellic acid	*Gibberella fujikuroi*	Rice, bran and malt residue	1300 mg/kg of substrate	[98]
Gallic acid	*Aspergillus niger*	Black Plum seed powder	14.5 mg/g of substrate with glucose as carbon source	[99]
Tannase and Gallic acid	*Aspergillus oryzae*	Fruit seeds of apple, guava, tamarind, black plum and watermelon	30 U/g for tannase and 16 mg/g for gallic acid	[100]
Fumaric acid	*Rhizopus arrhizus*	Soyabean cake	0.86 g/g	[101]
Citric acid	Aspergillus niger and Trichoderma reesei	Sugarcane bagasse	2.51 mg/g	[102]
^Chlorogenic acid^	Aspergillus niger	Coffee pulp	600 mg/kg of coffee pulp	^(^[103]
^Indole-3-acetic acid (IAA) and Conidial spore^	*Trichoderma harzianum*	grass clippings and pruning waste	101.46 µg *g*^−1^ dry matter IAA and 3.03 × 10^9^ spore *g*^−1^ dry matter	[104]^)^
^Butyric acid^	*Clostridium tyrobutyricum*	Wheat bran, rice polishings and molasses	5.63 mg/100 g from rice polishing	[105]
**^Antibiotics^**				
Natamycin	*Streptomyces gilvosporeus* Z28	wheat bran, rapeseed cake, rice hull and crude glycerol	9.27 mg.gds^-1^	[5]
Paclitaxel	*A. fumigatus TXD105*	Sugarcane bagasse	145.61 mg/kg	[106]
Monascorubrin	*Penicillium minioluteum ED24*	Sesame seed cake		[107]
Antioxident	*Aspergillus niger GH1*	Grapefruit by-products	7.64 mg/g at 96 h of fermentation	[108]
Lovastatin	*Aspergillus terreus*	Corymbia maculata Leaves	10.1 mg/g with fresh leaves and Lvs-r strain	[109]
^Hispidin^	*^Phellinus linteus^*	^Black rice and Pearl barley medium^	179.01 mg/kg dry weight pearl barley medium	^(^[110]
^6-pentyl alpha pyrone (6 pp)^	*^Trichoderma asperellum TF1^*	^Blend of jatropha cake, vine shoots, olive pomace & olive oil^	7.36 ± 0.37 mg g DM-1	[111]^)^
**^Enzymes^**				
Alpha amylase	*Aspergillus oryzae*	Edible oil cake	10,994.74 U/gds	[112]
Amylase	*Bacillus subtilis* D19	Wheat bran	1239 U/g	[113]
Lipase	*Aspergillus terreus* NRRL-255	Cacay oil and butter	2867.18 U g^-1^	[114]
Pectinase	isolated fungus VTM4	Coffee pulp	0.747 U/mL	[115]
Xylanase	*Aspergillus niger* CCUG33991	Wheat bran	2919 U/g	[116]
Protease	*Bacillus halodurans*	Wheat bran	401.18 U/ml	[117]
Beta glucosidase	*Aspergillus* sp. DHE7	Jojoba meal	232.6 U/mL	[118]
Alpha galactosidase	*Penicillium* sp.	copra mannan extract	6.672 U/ml	[119]
**^Biosurfactants^**				
Rhamnolipid	*Pseudomonas aeruginosa PTCC 1074s*	Soyabean meal	14.63 g/kg substrate	[120]
Biosurfactant	*Bacillus subtilis strain Al-Dhabi-130*	Crude oil	89 %of the crude oil	[121]
Sophorolipids	*Starmerella bombicola*	oil cake and molasses	0.2 g SL *g*^−1^	[122]
Rhamnolipid	*Pseudomonas aeruginosa*	sugarcane bagasse and sunflower seed meal		[123]
Surfactin	*Bacillus amyloliquefaciens*	Rice straw and soybean flour	15.03 mg/gds	[124]
Biopesticides				
Biopesticide conidial spore	*Trichoderma harzianum*	rice husk and beer draff	2.0 × 109 conidia g-1dm	[125]
Conidial spore	*Trichoderma asperellum*	vine shoots, jatropha cake, olive pomace and olive oil	8.55 ± 0.04 × 10^9^ conidia/g DM	[126]
Biopesticide spores	*Trichoderma Brev T069*	cassava peels	9.31 × 10^9^ spores/g of Cassava peel	[127]
^Fungal biopesticide^	*^Trichoderma harzianum^* *^Beauveria bassiana^*	^Rice husk^	m 2.0 × 108 to 2.0 × 109 spores g-^1^dr^y matter^	^(^[128]

### 5.3 ^Organic acids^

For organic acid synthesis, the environment should be controlled, including temperature, humidity, and ventilation. Such parameters as temperature, moisture content and acid concentrations are monitored for increased efficiency of organic acid production. Different types of organic acids can be synthesized by SSF, such as citric acid, lactic acid, gluconic acid, acetic acid, etc., depending on the substrates and microbes used [129]. The most important industrial organic acids produced by SSF are citric acid, lactic acid, and succinic acid. Citric acids have multiple industrial applications, and efforts have been made to enhance its production and reduce the cost through a fermentative approach. Agro-industrial wastes have been used for the production of citric acid, such as fruit waste (130), banana peel [131], apple pomace [132] and peat moss [42]. Fruit waste (pineapple and orange) was used for the citric acid production using *A. niger* strains NRRL 567 and 328, which resulted in 57.6 % and 55.4 % citric acid, respectively, with moisture content of 38.9 % [130]. An inexpensive substrate, namely banana peel, was used for citric acid production by SSF using *A. niger*; optimum conditions of 70 % moisture content, 28 °C temperature, pH 3, 10^8^ spores/ml inoculum and 72 h incubation time were determined for maximum production of citric acid [131]. Citric acid can be produced from apple pomace using *A. niger* NRRL 567, and 294.19 g/kg DS was obtained at optimum conditions [132]. In a study to produce succinic acid, fractionation of wheat was performed into bran, gluten and gluten free flour. Gluten free flour and gluten were hydrolyzed using solution containing enzymes to produce 140 g/L glucose and 3.5 g/L free amino nitrogen. Finally, SSF was used for the production of succinic acid using *Actinobacillus succinogenes* using glucose and amino nitrogen stream (hydrolysate), which resulted in 22 g/L succinic acid [79]. Pine needle as bed material was used as substrate for lactic acid production by solid state fermentation using several strains such as *Lactobacilli delbrueckii* (NCIM 2025), *Lactobacilli pentosus* (NCIM 2912), *Lactobacillus* sp. (NCIM 2734), *Lactobacillus* sp. (NCIM 2084), and a mixed culture of the former two strains; it was found that mixed culture attained the maximum lactic acid production of 45.10 g/L [133]. Other examples of recent organic acids produced by SSF are displayed in Table 1**.**

### 5.4 Bioremediation

SSF uses microorganisms grown on solid substrates to remove pollutants from the environment in the absence or almost absence of freely flowing water. Microorganisms, such as fungus or bacteria, grow and generate desirable chemicals which transform the contaminant into less hazardous form for the environment. Due to its versatility in handling different types of contaminants as well as its promise for sustainable and affordable solutions, this technique has attracted interest in environmental bioremediation. A variety of contaminants, including organic substances, heavy metals, and dyes, have been effectively treated using bioremediation through SSF. It is very helpful for cleaning up polluted sediments, soil, and some kinds of industrial waste. One benefit of SSF in bioremediation is that it may frequently be done on-site, minimizing the requirement for hazardous material transport and handling. However, choosing the right microbes, maintaining ideal conditions for microbial growth and activity, and maintaining the substrate are all crucial to the process's effectiveness. Before using this approach for significant bioremediation projects, careful study, testing, and consideration of site-specific elements are required. The eco-friendly approach was utilized by Kadam *et al.* to remove textile dye stuff by absorbing Red M5B dye, a dye mixture sample, and real textile effluent onto distillery industry waste yeast biomass (DIW-YB), followed by bioremediation using *Bacillus cereus* EBT1 under SSF [134]. Decolorization was observed after SSF which was confirmed by enzyme analysis. Metabolites after degradation was analysed by UV–Vis spectroscopy, FTIR, HPLC and GC-MS, where 98 % decolorization was observed by *B.cereus* in 36 h [134].

### 5.5 Biosurfactants

Surfactants are compounds having polarity and hydrogen bonds [135]. Natural surfactants are called biosurfactants possessing amphiphilic molecules with hydrophilic and hydrophobic fractions [136]. The polar groups can be ionic, non-ionic, amphoteric, and non-polar groups contain hydrocarbon chains. These properties of biosurfactant provide them with reduced surface and interfacial stress forming emulsion [137]. Biosurfactant are used for emulsification, detergent, lubrication and foaming. Thus, biosurfactant are used in industrial preparations such as cosmetics [138], laundry [139] and also in medical fields used as antimicrobial, antitumor and anti-inflammatory agent [140]. Food and Drug Administration (FDA) has approved production of biosurfactant using yeast and gave safe (GRAS) status [141]. Biosurfactant are a group of SM that can be divided into many groups based on the structure and source; these include glycolipids, natural lipids, lipopeptides, phospholipid, and polymeric surfactants. Rhamnolipids are glycolipid biosurfactant produced by *Pseudomonas* species. *Pseudomonas aeruginosa* PTCC 1074s fed with soyabean meal in the SSF for the synthesis of rhamnolipids at the optimum conditions (humidity 80 %, temperature 34.5 °C, inoculum size 1.4 ml, and glycerol 5 %) and was characterized by TLC, FTIR and H—NMR; results showed production of rhamnolipid of 19.68 g/kg dry substrate [120]. *Lactobacillus* and *Bacillus* strains grew in corn steep liquor produced by corn wet milling industry forming fermented medium to produce Lipopeptide and phospholipids biosurfactants which can be used in cosmetics and personal care industry [142].

### 5.6 Biofertilizer and biopesticides

Biofertilizer and biopesticide production through SSF is a sustainable and eco-friendly method of producing high-quality fertilizers using microbial activities on solid substrates. Chemically synthetic pesticides and fertilizers cause harm to the environment and humans. Thus, the production of biopesticides and biofertilizer have been developed for the biocontrol of harmful insect, pest and plants. Microorganism can produce toxins to compete or destroy the insects or pests. SSF was performed for the agro-industrial waste of cattle dung, vinegar production residue, and rice straw using *Trichoderma harzianum* for the production of bioorganic fertilizer to control fusarium wilt of cucumber [143]. *Trichoderma harzianum* was cultivated using two substrates with varying levels of biodegradability, rice husk and beer draff, in bioreactors ranging from 1.5 L to 22 L in capacity. This study introduces a sequential batch operation strategy (SBR) for fungal conidia production in SSF, aimed at enhancing the efficiency of conventional batch processes [125]. The production of conidia by *Metarhizium anisopliae* using SSF was carried out using rice as the substrate in plastic bags and tubular bioreactors. Strain conservation, reactivation, and propagation ensure genetic stability, with growth parameters monitored through CO_2_ production. Conidial yield exceeds 1 × 10⁹ conidia/g dry substrate after 10 days, with over 80 % germination and 75 % viability, resulting in more than 80 % insect mortality in bioassays using *Tenebrio molitor* [144]. Bioherbicide production from *Phoma* sp. using solid-state fermentation was optimized with bagasse, soybean bran, and corn steep liquor as substrates. The optimal conditions, including 70 % moisture, 30 % soybean bran, and 20 % corn steep liquor, resulted in a phytotoxicity level of 40 against target plants. The bioherbicide's mode of action involved inhibiting carotenoid biosynthesis [145]. Biomass production of *Purpureocillium lilacinum* KU8 on wheat bran under SSF was optimized by identifying key factors: moisture content, yeast extract, and incubation time. The optimal conditions were 67.98 % moisture, 2.29 % yeast extract, and 142.2 h of incubation, resulting in 107.46 mg/g of biomass, which demonstrated effective bio-nematicidal properties [146]. Kitchen waste was used as substrate for performing SSF to produce a *Bacillus thuringiensis*-based biopesticide. An orthogonal test was performed to determine the best composition for the culture medium, and results showed 55.21 % kitchen waste, 22.08 % wheat bran, 11.04 % soybean cake power, 11.04 % grain hulls, and 0.63 % mixed ions. This led to a spore count of 5.01 × 10^10^ CFU/g and entomotoxicity of 15,200 IU/mg. The procedure was scaled up effectively to a 35-kilogram level and showed potential cost advantages [147].

## 6 Summary

SSF represents a promising and sustainable approach to valorize agro-industrial waste by transforming it into valuable secondary metabolites. This review highlights the potential of SSF for converting organic-rich substrates, such as agricultural residues and industrial byproducts, into products like enzymes, antibiotics, organic acids, biofuels, biosurfactants, and biofertilizers. SSF's environmental and economic advantages, including reduced energy consumption, minimal contamination risks, and the ability to utilize waste as a low-cost substrate, underscore its relevance in advancing circular bioeconomy and waste management. However, challenges such as mass and heat transfer limitations, reactor design, and scale-up issues remain significant barriers to its commercial adoption. Moreover, integrating emerging technologies, such as genetic engineering and advanced bioreactor designs, could further enhance the efficiency and scalability of SSF. Continued research and technological advancements are essential to overcome existing challenges and unlock the full potential of SSF for large-scale applications, contributing significantly to sustainable development and waste valorization.

## 7 Challenges and future prospects

SSF is the means to produce biomolecules and secondary metabolites using agro-industrial waste as substrate for industrial and pharmaceutical purposes. With the advancement of technologies, SSF has led to the production of high-value products. The challenges associated with SSF include heat dissipation, mass transfer, and biomass estimation, which need to be addressed with an advanced bioreactor. Improving microbial strains through CRISPR CAS9 technology and genetic engineering can generate desired products in bulk for pharmaceutical industries, food industries, medicine, chemicals, agriculture, animal models and fuel production. CRISPR-Cas systems have enabled genome editing in multiple industrially relevant species and provided genetic tools to edit a genome through gene knockouts or homology-mediated knockins to control transcription of exogenous or endogenous genes. CRISPR-Cas-mediated engineering can increase the number of chemicals and products that are accessible through fermentation and broaden the diversity of strains suitable for industrial production. These technologies can enhance the SSF procedure as the factors influencing the product formation like moisture, temp, pH etc., may not be the limiting factor and thus, product formation can be enhanced. Compact design, less time consumption and yield in bulk still need to be achieved.

## CRediT authorship contribution statement

**Mohammad Perwez:** Writing – original draft, Methodology, Investigation, Formal analysis, Conceptualization. **Sameer Al Asheh:** Writing – review & editing, Methodology, Investigation, Formal analysis, Conceptualization.

## Declaration of competing interest

The authors declare no conflicts of interest regarding the publication of this paper. No financial or personal relationships exist that could have influenced the research presented. All authors have reviewed and approved the manuscript and confirm its integrity and accuracy. This paper represents the opinions of the author(s) and does not mean to represent the position or opinions of the American University of Sharjah.
